# New Era in the Treatment of Iron Deficiency Anaemia Using Trimaltol Iron and Other Lipophilic Iron Chelator Complexes: Historical Perspectives of Discovery and Future Applications

**DOI:** 10.3390/ijms22115546

**Published:** 2021-05-24

**Authors:** George J. Kontoghiorghes, Annita Kolnagou, Theodora Demetriou, Marina Neocleous, Christina N. Kontoghiorghe

**Affiliations:** Postgraduate Research Institute of Science, Technology, Environment and Medicine, 3021 Limassol, Cyprus

**Keywords:** maltol, ferric maltol, Feraccru, Accrufer, iron deficiency, iron deficiency anaemia, lipophilic chelators, alpha-ketohydroxyheteroaromatic chelators, pharmacology, clinical applications

## Abstract

The trimaltol iron complex (International Non-proprietary Name: ferric maltol) was originally designed, synthesised, and screened in vitro and in vivo in 1980–1981 by Kontoghiorghes G.J. following his discovery of the novel alpha-ketohydroxyheteroaromatic (KHP) class of iron chelators (1978–1981), which were intended for clinical use, including the treatment of iron deficiency anaemia (IDA). Iron deficiency anaemia is a global health problem affecting about one-third of the world’s population. Many (and different) ferrous and ferric iron complex formulations are widely available and sold worldwide over the counter for the treatment of IDA. Almost all such complexes suffer from instability in the acidic environment of the stomach and competition from other dietary molecules or drugs. Natural and synthetic lipophilic KHP chelators, including maltol, have been shown in in vitro and in vivo studies to form stable iron complexes, to transfer iron across cell membranes, and to increase iron absorption in animals. Trimaltol iron, sold as Feraccru or Accrufer, was recently approved for clinical use in IDA patients in many countries, including the USA and in EU countries, and was shown to be effective and safe, with a better therapeutic index in comparison to other iron formulations. Similar properties of increased iron absorption were also shown by lipophilic iron complexes of 8-hydroxyquinoline, tropolone, 2-hydroxy-4-methoxypyridine-1-oxide, and related analogues. The interactions of the KHP iron complexes with natural chelators, drugs, metal ions, proteins, and other molecules appear to affect the pharmacological and metabolic effects of both iron and the KHP chelators. A new era in the treatment of IDA and other possible clinical applications, such as theranostic and anticancer formulations and metal radiotracers in diagnostic medicine, are envisaged from the introduction of maltol, KHP, and similar lipophilic chelators.

## 1. Introduction

Normal biological, metabolic, physiological activities, and bodily functions are maintained in life due to the supply of essential nutrients, including transition metal ions, such as iron, copper, and zinc [1,2,3,4,5,6,7,8,9]. Healthy living is ensured following the acquisition of daily dietary requirements and maintaining a concentration of iron and other metal ions in the tissues. In contrast, iron and other metal metabolic imbalances are associated with serious clinical conditions [1,2,3,4,5,6,7,8,9,10,11]. In particular, one of these clinical conditions, namely iron deficiency anaemia (IDA), affects about one-third to a quarter of the world’s population, with outcomes including increased child and maternal mortality, pregnancy complications, cardiac complications, fatigue, reduced physical and mental performance, paleness, koilonychia, etc. [12,13,14,15]. In most of these cases, the symptoms of iron deficiency are cured using iron supplements, which are widely available.

Iron is found in all cells of the body. It is required for many essential bodily functions and physiological processes, including oxygen transport, storage, utilisation, and energy transduction [1,2,3,4,11].

Different mechanisms, pathways, and proteins are involved in the uptake, distribution, utilisation, recycling, and excretion of iron and other essential metal ions in humans, as well as other organisms. In this context, each cell requires and utilises different amounts of these metal ions for different biological functions [1,2,3,4,11]. The transfer of iron to all cells of the body is accomplished by transferrin present in blood and is mediated through transferrin receptors present on the cell membrane [11,16,17,18]. Intracellular iron storage is accomplished by ferritin and haemosiderin. One molecule of ferritin can store up to 4500 molecules of iron and haemosiderin is a cluster of ferritin molecules with a broken protein shell, which mainly predominates over ferritin in iron loaded conditions [11,19,20,21,22].

In relation to the human body, about 4.5–5.0 g of iron is estimated to be present and distributed in blood and different organs of a 70–75 kg average adult man. Most of the iron is in the ferrous state in a complex form with a protoporphyrin ring (haem), in the protein haemoglobin (2.3–2.6 g) found in red blood cells (RBC), and myoglobin (0.32–0.40 g) found in the muscle. The remaining iron is distributed in the body mainly in the form of polynuclear ferric oxyhydroxide phosphate complexes in the iron storage proteins ferritin (0.7 g) and haemosiderin (0.3 g) found mainly in the liver, spleen, muscle, and bone marrow. Iron is also found in other proteins, such as mitochondrial cytochromes (17 mg), catalase (5 mg), transferrin (4 mg), and non-haem iron containing enzymes (0.1 g) [11].

Under normal physiological conditions, iron is mainly found in the ferrous (Fe^2+^) or ferric (Fe^3+^) state forms, always bound to different ligands containing oxygen, nitrogen, and sulphur [11]. At physiological pH, ferrous iron in aqueous solutions is oxidised to ferric iron, which is only found in trace detectable levels, since it mostly precipitates by forming insoluble polymeric ferric oxyhydroxide complexes with a high stability constant (log K = 38) [11]. The solubility of iron and other metal ions increase at acidic pH including the acidic environment of the stomach.

Many molecules, including food components and drugs, contain metal binding ligands, which can form complexes with iron. The complexes being formed can affect the solubility, interactions, and transfer properties of iron across cells in the gastrointestinal tract and in other parts of the body [11,23].

Different pharmacological, toxicological, and therapeutic characteristics of iron complexes are observed in vivo, which depend on the size, solubility, lipophilicity, and stability of the complex, as well as other physicochemical parameters [11,24,25]. Furthermore, the characteristics of the iron complexes are also influenced by competing endogenous low molecular weight chelating molecules and iron chelating proteins, such as transferrin and lactoferrin [26,27]. Following these interactions and exchanges the chelator involved in the iron complex is, in most cases, dissociated, and the iron molecule enters the iron metabolic pathways. The chelator dissociated from the iron complex has different metabolic, toxicological, and pharmacological properties in comparison to the chelator iron complex [28].

Iron and other metal metabolic imbalances are associated with serious medical conditions such as IDA, which affect billions of the people worldwide [12,13,14,15]. Iron deficiency anaemia could be caused by many genetic, nutritional, metabolic, and other factors, as well as diseases [12,13,14,15,29,30,31,32,33]. Iron supplementation is used in food products, such as cereals or prescribed by physicians in most cases for the treatment of IDA, including many and different oral ferrous and ferric iron complex formulations, which are widely available and sold worldwide at pharmacies [13].

There are many safety concerns arising from the uncontrollable use of iron supplements, mainly because of the potential toxicity implications arising from the free radical catalytic and carcinogenic properties of iron. In general, only a small portion of orally administered iron is absorbed, and most of it is excreted in the faeces. The presence of excess iron is toxic to the cells of the gastrointestinal tract [34]. Furthermore, some iron complexes, including those with nitrites and haem components found in processed and red meat, respectively, are suspected to be major causes of colorectal and other cancers [35].

Despite the wide availability of oral ferrous and ferric iron formulations, and of intravenous iron formulations in the treatment of IDA, there is scope for further improvement, especially in relation to safer and more effective targeting, with the ultimate aim to control increased iron absorption and delivery to haematopoietic and other tissues. In this context, trimaltol iron (International Non-proprietary Name: ferric maltol, Feraccru or Accrufer), and other lipophilic chelator iron complexes appear to offer improved therapeutic advantages in the treatment of IDA conditions in comparison to traditional oral iron formulations, which are generally linked to gastrointestinal toxic side effects, and in some cases, complications associated with exacerbation of other pre-existing underline diseases [11,23,34,36].

## 2. Iron Absorption and Distribution Pathways

Under normal conditions, the absorption of iron in the gastrointestinal tract and the distribution pathways to the haematopoietic and other tissues are regulated and controlled by a number of proteins and transcription factors, as well as other genetic, environmental, nutritional, and other factors [1,2,3,4,11,23,36].

### 2.1. Iron Absorption in Humans

Iron absorption in humans under normal conditions is thought to be controlled by a number of regulatory proteins of iron metabolism, which in conjunction with other regulatory pathways of iron utilization and excretion leads to body iron balance.

In general, iron balance in the human body is maintained when the amount of iron absorbed is equivalent to the amount of iron lost through excretion and other routes [23]. This dynamic balance is usually controlled and operated within limitations provided by the regulatory pathways and are mostly influenced by dietary iron and the haematopoietic activity [1,2,3,4]. Minor changes in iron balance can usually be restored at a different rate, depending on the level of change in the iron stores. For example, in blood donors or long distance runners iron loss is gradually restored from increased dietary iron absorption [37,38]. However, imminent blood transfusion is required following excessive bleeding, for example, after motor accidents or surgery, where there is substantial loss of blood and iron, as well as limited capacity in rapidly restoring these losses. Similarly, iron balance is restored when, for example, excess iron is gradually lost from patients who receive iron supplements long-term, or a small number of transfusions [23].

In contrast, iron imbalance could also be caused and maintained in conditions where the rate of iron absorption may be greater than the rate of iron loss, as shown in African siderosis (Bantu siderosis), or where the rate of iron loss may be greater than iron absorption, as shown in vegetarian populations with IDA or in malnutrition, which is caused by insufficient intake of dietary iron [23]. In addition to genetic and regulatory factors, many nutritional, environmental, and other factors could also influence the rate of iron absorption and body iron load as shown in Table 1. In particular, the quantity and quality of iron entering the gastrointestinal tract could play a significant role in the overall absorption rate of body iron intake. Furthermore, dietary habits, such as the level of alcohol and water intake, as well as different food types and drugs, could also influence this process (Table 1) [23].

In general, vegetarian meals low in dietary iron are likely to reduce the intake of iron and rate of body iron load in vegetarian populations [39,40,41]. In contrast, in meat eating populations, sufficient amounts of iron are absorbed because of the presence of high concentration of haem, which is the main form of iron to be found in the meat dishes. Haem is a lipophilic iron complex with a protoporphyrin ring, which is better absorbed from the gastrointestinal tract than other forms of dietary iron [11].

### 2.2. The Role of Proteins of Iron Metabolism in the Regulation of Iron Absorption

Iron uptake from the gastrointestinal tract is regulated by several mechanisms and specific proteins. Modifications or abnormalities in the regulatory mechanisms can cause changes in the rate of iron absorption and overall body iron load. For example, increased iron absorption is observed in genetic diseases, such as hereditary haemochromatosis, and thalassaemia intermedia leading to iron overload in both conditions [42].

**Figure 1:**
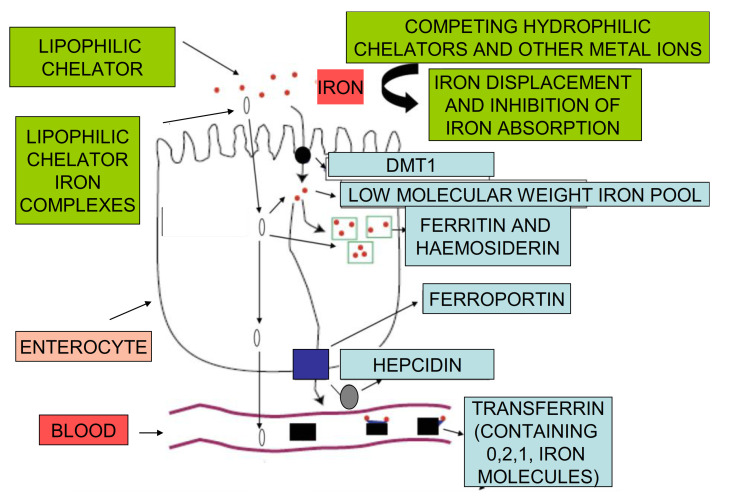
Mechanisms of iron absorption at the enterocyte. Animated image of the regulatory pathways involved in iron absorption. Iron metabolic pathways involving the apical divalent metal transported protein (DMT1), ferritin, hepcidin, ferroportin, and transferrin. A parallel, non-regulatory iron absorption pathway is also shown using lipophilic iron chelator complexes. In contrast to lipophilic chelators, hydrophilic chelators appear to inhibit iron absorption.

Under normal conditions there are several proteins and transcription factors that appear to play an important role in controlling the rate of iron transfer from the gastrointestinal tract to the blood stream. The regulatory control of iron absorption and utilization in the enterocyte includes several steps and pathways, which appear to involve a number of proteins such as ferrireductase(s), the apical divalent metal transported protein (DMT1), ferroportin, hepcidin, transferrin, and ferritin (Figure 1) [1,2,3,4,42,43,44,45,46].

The initial step of the mechanism in the regulatory pathway of iron absorption is thought to involve the reduction of dietary ferric iron to ferrous iron by a ferrireductase at the surface of the enterocyte before its intracellular transport by DMT1 (Figure 1) [1,2,3,4]. Following intracellular entry in the enterocyte, iron is partly incorporated into ferritin or transferred into the low molecular weight, intracellular iron pool. In subsequent steps, iron is transported to ferroportin at the basolateral membrane of the enterocyte, which then exports it in plasma, where it is bound by transferrin. Iron bound by transferrin is then transferred to all of the cells of the body via an uptake mechanism by transferrin receptors present at the cell surface, followed by intracellular dissociation of iron from transferrin in an endosome of acidic pH [1,2,3,4].

Hepcidin, a protein hormone produced by the liver, is believed to play a major role in iron absorption [43]. Hepcidin is a key regulator of iron metabolism, controlling the release of iron into the circulation. In relation to iron absorption hepcidin can prevent the release of iron into plasma by binding ferroportin at the basolateral membrane of the enterocyte, causing its internalization and degradation within the enterocyte [43,44,45,46,47,48]. As a result, iron is not released into plasma, but remains trapped in the enterocytes and returns in the gut lumen since enterocytes are shed every 2–3 days. Several other pathways of iron metabolism could be affected by hepcidin, including the inhibition of iron release into plasma from macrophages [1,2,3,4,43]. Furthermore, these effects and also other related effects could influence the overall rate of iron loading and distribution in the body, including the haematopoietic tissues, leading among other things to iron metabolic imbalance conditions, such as IDA, the anaemia of chronic disease, and iron overload [1,2,3,4,42,43,44,45,46,47,48].

Iron absorption is also affected by changes in other regulatory molecules in addition to hepcidin. In particular, the expression of ferroportin and DMT1 appear to be affected by several other factors and to be different in the duodenum, haematopoietic tissues, liver, kidney, and also other organs [43,44,45,46,47,48,49]. In this context, several factors are involved, including the signal to the intestine to increase iron absorption, which appear to be influenced by regulatory molecules, which sense the iron stores [1,49,50]. Similarly, different regulatory mechanisms appear to control the production of haemoglobin in the haematopoietic tissues and to prevent anaemia. In this context, the regulatory peptide erythroferrone and the hormone erythropoietin play a key role in erythropoiesis and the regulation of iron absorption [1,2,4]. Overall, it appears that, in these cases, the influence of the regulatory molecules cause an increase in the expression of DMT1 and a corresponding increase in the uptake of iron [1,49,50].

### 2.3. Differences among Individuals in Iron Absorption Requirements

The daily requirements for iron differ among individuals and depend on several parameters including age, sex, life style, sport activity, health status, etc. [15,29,30,31,32]. Most of the iron present in the body, which is estimated to be about 4–5 g in normal adults, is conserved and recycled. Of particular importance to iron metabolism and especially the haematopoietic activity, is the recycling of iron present in haemoglobin, which amounts to more than 60% of the total body iron content [1,4,11,23]. In contrast, only a few milligrams of iron are excreted or lost and these are replaced from dietary iron, which is a small component of variable amounts in different foods. However, it is envisaged that small loses of iron and insufficient intake of dietary iron over long periods will result in iron deficiency.

Under normal conditions, the absorption of iron in a western diet is generally estimated to be about 2 mg/day and equivalent losses allow the maintenance of body iron balance. However, there are many different dietary variations between individuals and also populations worldwide (Table 1) [11,23].

Nutritional studies have suggested some general estimation for the daily iron requirements in different categories or groups of people. In adult men and post–menopause women, the daily requirement is about 8 mg, which is generally considered to be low in comparison to other groups. The highest daily requirement for iron is for pregnant women, which is about 27 mg, whereas for adult women is 18 mg, and for breastfeeding women, 9–18 mg. In comparison, the daily requirements of iron for teenage boys and girls is estimated to be about 11 and 15 mg, respectively [29,30,51].

Despite the differences in the daily iron requirements in each category, and each individual, the rate of body intake of iron could be affected by many other factors, and their combinations, including genetic predisposition, the erythropoietic activity of the bone marrow, the quantity and quality (haem or non-haem) of dietary iron, the presence of other dietary components, such as reducing agents, phytochelators, natural chelators, chelating drugs, and many others (Table 1) [29,30,31].

## 3. Non-Regulatory Mechanisms of Iron Absorption

Despite that major emphasis on the mechanisms of iron absorption is mainly associated with the regulatory role of proteins of iron metabolism, the overall rate of iron absorption is also governed by many dietary and other factors. Furthermore, the capacity of the protein regulatory pathway is limited and may not be effective under certain conditions.

### 3.1. Dietary Molecules and Forms of Iron Affecting Its Absorption

**Figure 2:**
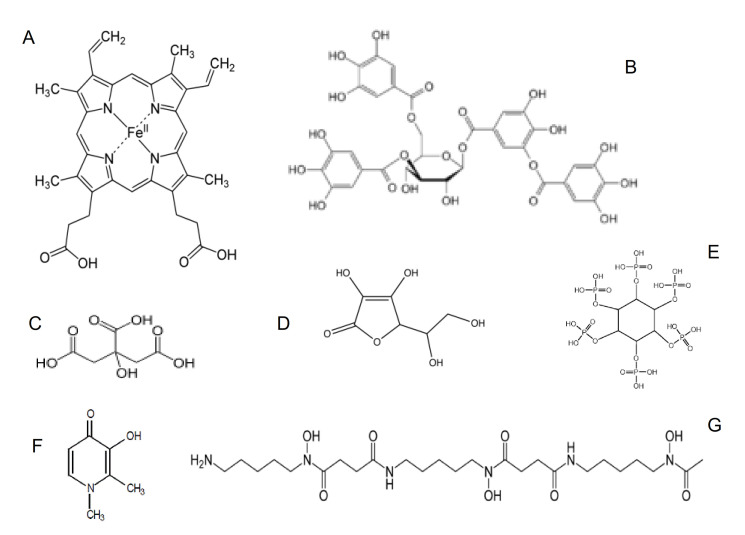
The chemical structure of molecules involved in iron absorption. Haem iron (**A**) is present in meat products and is better absorbed than non-haem iron. Tannic acid (**B**) and phytic acid (**E**) inhibit non-haem iron absorption. Citric acid (**C**) and ascorbic acid (**D**) facilitate non-haem iron absorption. The chelating drugs deferiprone (**F**) and deferoxamine (**G**) inhibit iron absorption.

There are many dietary variations among individuals, groups, and populations, particularly in relation to iron availability in the consumption of meat or vegetarian foods. Haem iron is much better absorbed than other forms of iron and it is the main source of dietary iron found in meat containing foods, which predominate in western diets (Figure 2) [41]. In contrast to western populations, the most severe pathological complications in relation to IDA are observed in the malnourished and vegetarian populations of developing countries. In the latter populations, haem iron in the diet is, by comparison, much lower, and overall iron absorption is not in significant amount from other dietary forms found in vegetarian meals [23,36,37,38,39,40,41].

Overall, it appears that in vegetarian and malnourished populations the rate of iron loss is higher than the rate of iron absorption from the iron present in vegetarian meals, resulting in a negative iron balance and, in the long-term, in iron deficiency [23].

One of the major factors influencing the rate of iron absorption is the apparent solubility of iron at the enterocyte site or possibly in other sections of the gastrointestinal tract, where iron may also be absorbed under different conditions (Table 1). The solubility of ferric iron in aqueous solution at physiological pH is negligible (10^−18^ mol/L) and iron precipitation rapidly occurs in biological fluids in the absence of low or high molecular weight naturally occurring chelators, such as citric acid and transferrin, respectively (Figure 2) [11].

In general ferrous iron is more soluble than ferric iron under the same conditions and is more readily absorbed. In general, ferrous iron forms or ferrous formulations, and the presence of reducing agents, such as ascorbic acid, will cause an increase in the solubility and overall absorption of iron from the enterocyte in comparison to most other ferric iron forms or formulations (Figure 2) [11,51,52]. In contrast, dietary molecules, such as tannins, phosphates, and other natural chelators or drugs, causing the precipitation of iron, will reduce the rate of iron absorption (Figure 2).

The interactions of iron with dietary molecules or drugs and their effects on iron absorption from the gastrointestinal tract could also be influenced by other essential or xenobiotic metal ions competing with iron [11,23,28,53].

### 3.2. Iron Chelating Drugs and Other Drugs Affecting Iron Absorption

Many orally administered drugs, including iron chelating drugs, interact with iron in the gastrointestinal tract and affect iron absorption. In this context, the hydrophilic iron chelating drugs, deferiprone and deferoxamine, when used orally (Figure 2F,G) form hydrophilic chelator iron complexes, which do not facilitate the transport of iron in the enterocyte and other cells, and generally inhibit iron absorption (Figure 1) [53,54,55,56]. In particular, deferoxamine is widely used in the prevention of iron absorption in accidental iron poisoning [55,56].

The iron chelating drugs are widely used for the treatment of iron overload by increasing iron excretion in iron overloaded patients [57,58]. It is envisaged that dietary molecules with properties similar to deferiprone and deferoxamine will not only cause a decrease in iron absorption when administered orally, but also an increase in iron excretion and an overall negative iron balance. The prolonged administration of iron chelating drugs in non-iron loaded individuals is expected to cause a reduction in the body iron stores and low availability of iron to the haematopoietic tissues, leading to IDA.

There are many other orally administered drugs that can bind iron, but with lower affinity than the iron chelating drugs deferoxamine and deferiprone; in most of these, inhibition of iron absorption is expected including the cases of tetracycline, minocycline, and hydroxyurea. In addition, the absorption and bioavailability of these and other similar drugs, which form complexes with iron is also affected [59,60].

The interaction of dietary molecules and drugs with chelating properties on the absorption of iron has not yet been fully investigated. However, in these cases, competition for iron between dietary molecules and drugs is suspected. Furthermore, the possibility of formation of ternary mixed iron complexes between these molecules will also likely affect the absorption of iron [61].

### 3.3. Quantitative Aspects of Iron Affecting the Rate of Iron Absorption

A major factor affecting the rate of iron absorption in humans is the quantity of soluble iron forms present in the gastrointestinal tract (Table 1). In general, the amount of iron absorbed is to some extent proportional to the concentration of iron in food. This effect can be highlighted from some unusual cases of consumption of excess amount of iron. For example, African siderosis in rural Africa, the use of iron cooking utensils for the preparation of traditional sorghum-based beer led to progressive iron overload in a large number of individuals [62]. The possibility of excess iron absorption been caused by the presence of a genetic component or the high iron content of the traditional sorghum-based beer has also been considered. Similar cases of acute iron overload and toxicity from increased iron absorption is observed mainly in children as a result of accidental iron poisoning. In these cases large amounts of iron are rapidly absorbed, causing serious toxic side-effects that are sometimes fatal [63,64].

It appears that with the increased iron uptake observed both in African siderosis and accidental iron poisoning, the normal regulatory pathways involving hepcidin and ferroportin are overwhelmed and unable to influence or control the increased rate of gastrointestinal iron absorption and associated toxicity [62,63,64]. Similar questions have been raised for the role of hepcidin and ferroportin in the slow reduction of excess iron in iron loaded transplanted thalassaemia patients, who have not received any form of iron chelation therapy [52,65,66].

## 4. Iron Formulations Used for the Treatment of Iron Deficiency Anaemia

Many iron formulations are available and sold over the pharmaceutical counters worldwide for the treatment of IDA. The wide variety and selection of iron formulations is partly indicative of the commercial interest related to the large number of iron deficient patients worldwide, as well as the continuous efforts for the search and development of new, more effective, and less toxic iron complexes for IDA treatment. Usually the oral iron formulations are in a tablet, capsule, extended release tablet or capsule, or liquid preparations, which contain about 30–100 mg of elemental iron.

The development of new and more effective iron formulations is also related to the increased and more selective requirements for more specific iron formulations by an increasing number of patients in addition to IDA, with other categories of anaemia not related to increased body iron requirements, such as pregnant women or vegetarian populations (Table 2) [52]. Many of these other categories of anaemic patients have a different pathophysiology and tolerance to iron formulations by comparison to IDA patients (Table 2) [13,67,68].

One major category involving millions of patients are those suffering from the anaemia of chronic disease or anaemia of inflammation, including patients with inflammatory, infectious and neoplastic diseases, such as different cancer types and inflammatory bowel disease. In most of the anaemia of chronic disease cases, sufficient iron is stored in the body, but compartmentalised in the reticuloendothelial system and cannot become readily available to the haematopoietic tissues for the production of sufficient amounts of haemoglobin [67,68].

There are many and different iron formulation products available worldwide for the treatment of IDA and other anaemias. Despite the large number and long term experience with most of the available formulations, there is no satisfactory treatment in many cases and always a scope for improvement in the treatment of different categories of iron deficient patients. In this context, new patented iron formulations appear in the pharmaceutical markets at regular intervals claiming improved response in patients.

The risk/benefit assessment for the use of different iron formulations including ferrous, ferric, oral, intravenous, and slow release, in each disease category shown in Table 2 has not yet been fully examined or clarified. Furthermore, there is no general consensus among physicians in different countries for the use of any specific iron category of the available iron formulations.

However, the large selection of iron formulations can benefit patients experiencing toxicity with one of them, such as in the cases of gastric irritation, or low efficacy in iron absorption. In these cases new iron formulations can be selected and prescribed for better tolerance or higher efficacy. Furthermore, the selection of iron formulations in most European and other countries is subject to budgetary controls in public health institutions and the cheaper available product is usually selected by comparison to new patented iron formulations, which are, in most cases, very expensive [69].

Most of the ferric and ferrous iron formulation complexes used in IDA patients are based on naturally occurring sugar derivatives, which in general appear to partly increase the solubility of iron and facilitate its absorption from the gastrointestinal tract (Table 3) [70].

Several other non-sugar iron derivatives have also been used successfully for many years for the treatment of IDA (Table 3). Ferrous sulphate is one of the classic, non-expensive iron formulations, which has been widely used worldwide with satisfactory results in many patients [71,72]. Many investigators are also proposing the use of ferrous ascorbate for the treatment of IDA because of its high efficacy and low toxicity [73,74,75,76,77].

A new approach in the design and development of more effective and less toxic formulations containing iron complexes for IDA patients of different categories, is the use of iron trimaltol and other lipophilic iron chelator complexes, which were proposed many years ago, but only recently received approval for clinical use [25,78].

## 5. The In Vitro Properties of Iron Maltol and Other Lipophilic Iron Complexes

Many ferrous and ferric formulations are widely used and sold at pharmacies for the treatment of IDA, but most of these have a low therapeutic index and are not satisfactory because of low efficacy, and are frequently non tolerated, leading to poor compliance (Table 3). The concept of the introduction of new, more specific iron formulations with a higher therapeutic index, including the use of iron maltol and other lipophilic iron chelator complexes for the treatment of IDA, was initiated about 40 years ago [25]. In particular, the trimaltol iron complex was originally designed, synthesised, and screened in vitro and in vivo in 1980–1981, by one of the authors (G.J.K.), following his discovery of the novel alpha-ketohydroxyheteroaromatic (KHP) class of iron chelators (1978–1981), which were intended for clinical use in iron overload and other iron metabolic disorders, including the treatment of IDA [25]. The slow progress in the development of iron maltol and other lipophilic iron chelator complexes appears to be related to commercial considerations [69,79].

### 5.1. Physicochemical Properties of Lipophilic Chelators and Their Iron Complexes

An original screening system for identifying investigational new drugs (IND) for the treatment of iron metabolic disorders, including iron overload and IDA, was previously tested using a large number of known and new chelators and chelator iron complexes [25]. Many in vitro and in vivo procedures were involved in the screening process, including the physicochemical characterisation of the chelators and their iron complexes. In general, the physicochemical properties, such as the size, charge, and lipid/water partition of molecules affect their mode of action, including gastrointestinal absorption, membrane transport, and tissue distribution. In particular, IND of small size molecular mass, neutral charge, and high lipophilicity appear to facilitate passive transport across cell membranes.

Within this context, several physicochemical parameters of IND and their metal complexes could be investigated and predict, to some extent, related pharmacological activity. These parameters include the affinity of the chelators for iron and other metals, the stability and stoichiometry of their complexes at acidic and physiological pH, the solubility, charge, the lipid/water partition coefficients of the chelators and their iron complexes, etc. [25,57]. Examples of some physicochemical properties of chelating drugs, phytochelators, and other synthetic chelators, as well as their iron complexes, are shown in Table 4.

There is wide variation in the physicochemical parameters of both the chelators and their iron complexes. The differences between each chelator reflected in the parameters are the results of the structural features, metal binding ligands, and other side chains or substituents in the main structure of the chelator molecules. Similar differences in physicochemical parameters, including the charge, partition coefficient, and molecular mass are also observed among the chelator iron complexes (Table 4) [25,57].

All the chelators listed in Table 4, including maltol and deferiprone, form a 3:1 stoichiometry chelator: iron complex at physiological pH, with exception of deferasirox and deferoxamine, where 2:1 and 1:1 stoichiometry complexes are formed, respectively (Figure 2).

### 5.2. In Vitro Properties of Maltol and the Maltol Iron Complex

**Figure 3:**
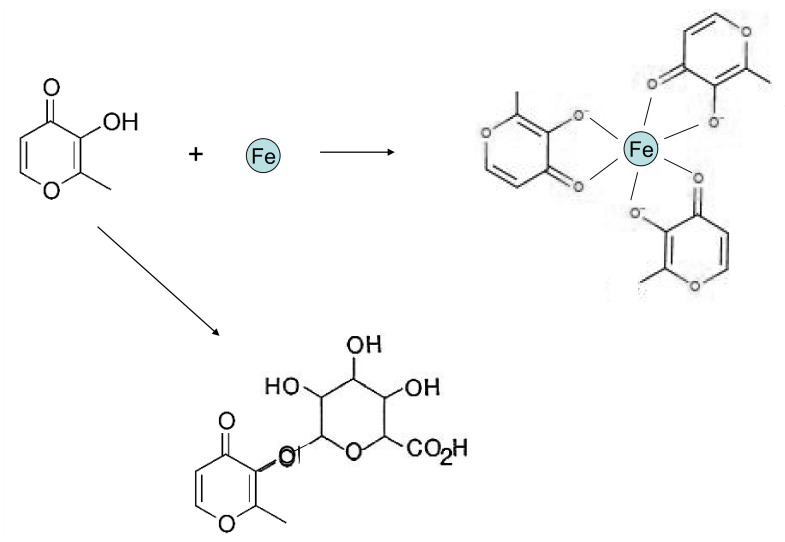
The formation of the tris-maltol iron (III) complex and maltol glucuronide. At physiological pH, maltol reacts with iron (III), forming the tris-maltol iron octahedral complex with iron in the centre. During iron (III) binding, a proton is displaced from the hydroxyl group of each maltol molecule, forming a negatively charged molecule, which coordinates with iron (III) of 3+ charge, forming a neutral trimaltol iron (III) complex. In humans, maltol is mostly metabolised to the maltol glucuronide conjugate, which has no iron or other metal chelating capacity.

Maltol is a naturally occurring alpha-keto hydroxy pyrone compound found in plants, e.g., in the bark of the larch tree and in pine needles [80]. It is also found in roasted malt and in bread, and is formed during caramelization. Maltol has been widely used and marketed for more than 50 years as a flavour enhancer and as a food additive. It is rapidly and extensively absorbed from the gastrointestinal tract (similar to ethyl maltol—an analogue of maltol) and both are mostly metabolised to glucuronide conjugates [81] (Figure 3).

Maltol was first identified as a potential iron chelator during the period of the four-step synthesis of the iron chelating drug deferiprone, where it was used as the starting material [25]. It was included in the list for the in vitro and in vivo screening procedure of natural and synthetic chelators intended for potential clinical use in iron overload, iron deficiency, and other diseases of iron metabolism [25,57]. The alpha-keto hydroxy iron binding site of maltol is identical to that of the chelating drug deferiprone (Figure 2F).

The in vitro screening procedure of the chelators involved the study and characterisation of iron complex formation, the interaction with proteins of iron metabolism, and the iron transport effects in red blood cells (RBC), as well as the iron transport effects in rat jejunum permeation [25,57].

**Figure 4:**
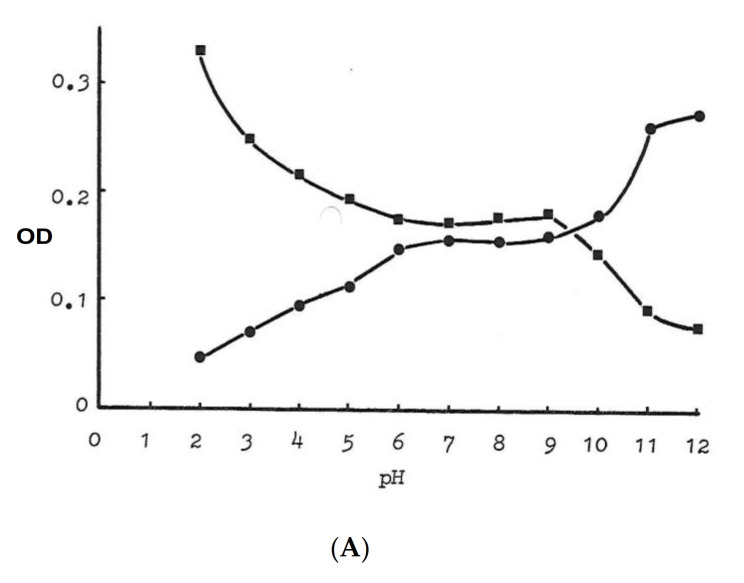

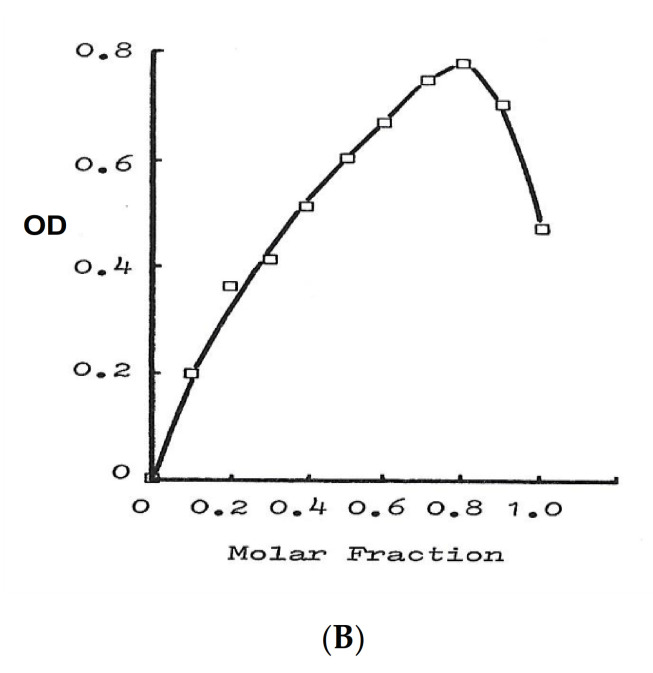
Characterisation of the maltol iron complex. (**A**) Stability of the maltol iron complex over a wide pH range. Titration of a mixture of maltol (0.75 mM) and iron (0.25 mM) at a pH range 2–12, indicating one iron complex species of red/orange colour at the pH 6–10 range. The optical density (OD) monitoring was carried out at the wavelengths of 320 nm (circles) and 280 nm (squares) [25]. (**B**) Identification of the stoichiometry of the tris-maltol iron complex at physiological pH. Estimation of the stoichiometry of the maltol iron complex using the Job’s plot method. Measurement of the optical density (OD) at 220 nm of different molar fraction mixtures of maltol (1 mM) and iron (1 mM) at pH 7.0. The horizontal axis refers to the molar fraction of maltol.

In the iron binding studies, a 3 maltol:1 iron stoichiometry complex (trimaltol iron) was formed at physiological pH using the Job’s plot method as indicated in Figure 4B [25]. While maltol is a white solid, forming a colourless solution, the iron complex of maltol is of deep red/orange colour, and appears stable at a wide pH range, including the pH 5–9 range, as shown in Figure 4A [25].

An octahedral structure iron complex similar to deferiprone and other bidentate chelators is formed in the interaction of maltol with iron at physiological pH (Figure 3 and Figure 4). A proton is released from the coordinating hydroxyl group of maltol on binding ferric (3+) iron, forming three negatively charged maltol molecules (Figure 3 and Figure 4) and an overall neutral trimaltol ferric iron complex. Both maltol and the maltol iron complex are lipophilic and have a neutral charge at physiological pH (Table 4).

### 5.3. Interactions of Maltol and the Maltol Iron Complex with Proteins

The pharmacological activity of maltol and the maltol iron complex is affected by a variety of proteins, including proteins involved in iron metabolism [28,57]. In particular, the interaction of maltol and the maltol iron complex with the iron transport protein transferrin and the iron storage proteins ferritin and haemosiderin is a major parameter characterising the mode of action of chelators, and is also a key part of the chelator evaluation for clinical use in diseases related to iron metabolism [19,20,21,22,24,25,28,57,82,83,84,85].

**Figure 5:**
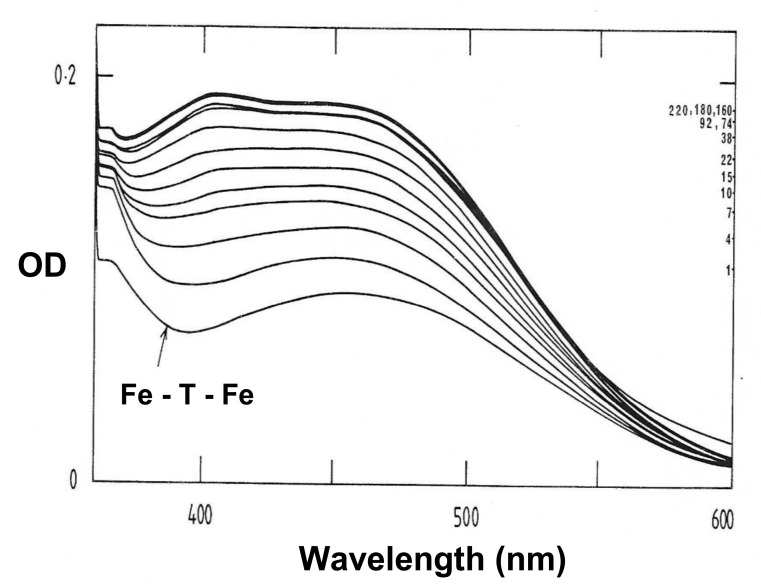
The interaction of maltol with iron saturated transferrin. Optical density (OD) spectral changes of the reaction of maltol (1.0 mM) with diferric transferrin (Fe-T-Fe) (0.036 mM) as a function of time from 1–220 min [25].

Substantial differences were observed in the original studies of iron mobilisation from diferric transferrin using maltol and other KHP chelators in vitro [25,86]. The 59-Fe radioactive labelled iron transferrin equilibrium dialysis studies have indicated that maltol is not effective in the mobilisation of iron from diferric transferrin. For example, in four different experiments for up to six hours, iron release from 59-Fe radioactive labelled diferric transferrin using deferiprone (4.0 mM) was 77–90%, and using maltol (4.0 mM) was 4–23% at the same conditions [25,86]. More iron was released from transferrin by maltol at 24 h incubations. However, UV-visible spectroscopic changes have also suggested that a ternary complex is formed between transferrin, iron, and maltol, but the maltol iron complex is not readily dissociated from transferrin (Figure 5) [25,86].

Similar results were obtained in iron mobilisation studies from diferric lactoferrin. For example, in equilibrium dialysis studies using 59-Fe radioactive labelled diferric lactoferrin iron release at pH 7.3 using deferiprone (0.29 mM) was 87.4%, and using maltol under the same conditions was 9.4% [87].

Under normal conditions, transferrin iron saturation in humans is only about 20–30%, with apo-transferrin, the most prominent species in blood by comparison to the two monoferric transferrins and diferric transferrin [17,18]. Apo-transferrin has high potency in mobilising mononuclear and low molecular weight iron, thus preventing microbial growth and the catalytic production of toxic free radicals, both requiring the presence of iron [88].

Apo-transferrin also has the capacity to mobilise iron from low molecular weight iron complexes, including iron from citrate, deferiprone and maltol iron complexes in plasma [88,89,90]. In this context, maltol or deferiprone iron complexes facilitate the transfer of iron to apo-transferrin, forming diferric and monoferric transferrin, which are then taken intracellularly through transferrin receptors in haematopoietic and other tissues [91,92,93,94,95].

In the initial studies of the interaction of maltol with ferritin, it was shown that maltol (1–4 mM) was effective in the mobilisation of ferritin iron (0.6–1.8 mM) at a slow rate reaction, which reached about 3 days to completion. It was estimated that the amount of iron released from ferritin by maltol (1.0 mM) at the end of the reaction was 24% by comparison to deferiprone 46%, used under the same conditions [25,96]. In further studies with maltol and other chelators, iron mobilisation from ferritin was confirmed and was also extended and shown to take place from other polynuclear forms of iron, such as haemosiderin and freshly prepared iron precipitates [97]. The rate of iron solubilisation from polynuclear forms of iron was higher in freshly prepared iron precipitates by comparison to haemosiderin and less so from ferritin [97]. Furthermore, less iron has been shown to be mobilised by maltol and other chelators from ferritin and haemosiderin possessing lower amounts of iron [98].

Similar results were observed in equilibrium dialysis 24 h studies using 59-Fe labelled horse spleen ferritin (0.15 mg/mL) containing 2200 molecules of iron per ferritin molecule and different chelators (1.2 mM). Under these conditions maltol caused the release of 9% of iron by comparison to deferoxamine 12% and deferiprone 21% [99].

The interaction of maltol and the maltol iron complex has also been investigated in other proteins of iron metabolism. In particular, maltol in a comparative study with the iron chelating drugs deferoxamine and deferiprone, as well as other compounds has been shown to inhibit the iron containing proteins lipoxygenase and cyclooxygenase, and associated metabolic pathways, such as thromboxane A2 synthesis and the conversion of arachidonate to HETE and HPETE in platelets [100,101,102]. The inhibitory effect of maltol in both cases was concentration dependent, reversible in the addition of iron, and lower by comparison to deferiprone and deferoxamine [100,101,102]. In the case of the thromboxane A2 inhibition, the median inhibitory concentration (IC50) was about 8 mM for maltol, 0.7 mM for deferoxamine, and 0.2 mM for deferiprone. Similarly, in the conversion of arachidonate to HETE and HPETE was 8 mM for maltol, 1.2 mM for deferoxamine, and 1.6 mM for deferiprone [101,102].

Overall, it appears that the interactions and effects of maltol with proteins of iron metabolism are concentration dependent and involve several components. In particular, maltol has the ability to mobilise intracellular low molecular weight iron, ferritin, and haemosiderin iron forming trimaltol iron complexes. Furthermore, trimaltol iron complexes can interact and donate iron to apo-transferrin and form monoferric and diferric transferrin, which subsequently can release iron to cells for storage, haemopoiesis, or utilisation for other cellular functions.

### 5.4. The Antioxidant Effects of Maltol and Other Iron Chelators

Iron is the major catalyst of free radical reactions and oxidative stress toxicity in biological systems. Many factors influence the rate of free radical reactions catalysed by iron in biological systems, including the pH, the presence of reducing agents, and of chelating agents [103,104,105,106].

The pro-oxidant and antioxidant effects of drugs are major parameters characterising their mode of action, as well as their toxicological and pharmacological properties. The modulation of free radical toxicity is more sensitive in the presence of iron and copper chelating drugs, which can strongly influence the catalytic activity of these metals [106,107,108,109].

The first antioxidant studies in relation to maltol have been carried out in 1986 [110]. Maltol and other chelators intended for clinical use have been tested in an original screening system involving three different models of free radical toxicity. In the first model, the iron induced free radical damage on the sugar component of DNA, deoxyribose, was carried out by monitoring malondialdehyde formation. Inhibition of deoxyribose damage was concentration dependent and ranged 1–4 mM in the case of maltol (49–80%) and 1–2 mM in the case of deferoxamine (90–91%) and also deferiprone (53–80%) [110]. In contrast, EDTA promoted free radical formation and caused an increase in deoxyribose damage at the same range of concentration (1–4 mM) and under the same conditions. In another model of free radical damage using UV irradiation on IgG, which resembles the formation of immunocomplexes of IgG in inflammation, the inhibitory effect of maltol (0.05–0.1 mM) was moderate (3–22%). In the third model of lipid peroxidation, the breakdown product malonaldehyde was monitored in mouse skeletal muscle homogenates resembling tissue damage, which is observed in many clinical conditions, such as muscular dystrophy, thalassaemia, and cancer. Under these conditions, maltol (0.5 mM) has been shown to exert antioxidant effects and to cause 79% inhibition [110]. Similarly, 13 other chelators have also been tested causing variable inhibition ranging from 99% maximum with deferiprone and 17% minimum with 8-hydroxyquinoline [110].

Following the above findings, the antioxidant activity of maltol has also been studied in several other free radical models [99,100,101,110]. Further studies followed where maltol as a food component or flavour enhancer has also been identified to possess potent antioxidant properties [111,112,113,114,115,116]. The potential application of maltol as a therapeutic antioxidant has also been identified in several disease models, including the treatment of inflammatory conditions, colitis, hepatotoxicity, diabetic peripheral neuropathy, carbon tetrachloride poisoning, etc. [117,118,119,120,121,122].

Studies on the pro-oxidant effects of iron complexes have also confirmed that ferrous sulphate, but not the trimaltol iron complex, promotes lipid peroxidation [110,123]. In general, it appears that maltol under physiological conditions can inhibit the catalytic formation of free radicals and lipid peroxidation caused by iron, and that the trimaltol iron complex does not exhibit pro-oxidant effects [99,100,101,110,111,112,113,114,115,116,123].

## 6. Cell Studies Using Maltol and Other Chelator Iron Complexes

The transport of chelators and chelator iron complexes across cell membranes provides important pharmacological information and is an important parameter in the screening procedure for use in iron metabolic diseases, including IDA.

The interaction of chelator iron complexes with transferrin and utilisation of iron from the chelator iron complexes by haematopoietic and other cells for the production of haemoglobin are major factors contributing to the efficacy of iron formulations intended for the treatment of IDA [16,25,42].

Iron transfer, donation, utilisation, and exchange differ among chelator iron complexes and various cell types, including cell lines. Within this context, different cell studies have been initiated in the original screening system and continue until today for clarifying the mechanisms and mode of action of the maltol iron complex in the treatment of IDA [25].

### 6.1. Iron Transport by Maltol and Other Chelators in Matured Red Blood Cells

The identification of the suitability of the maltol iron complex for the treatment of IDA was first realised in matured RBC experiments by measuring the incorporation of iron following incubation of RBC with maltol iron and other chelator iron complexes [25].

**Figure 6:**
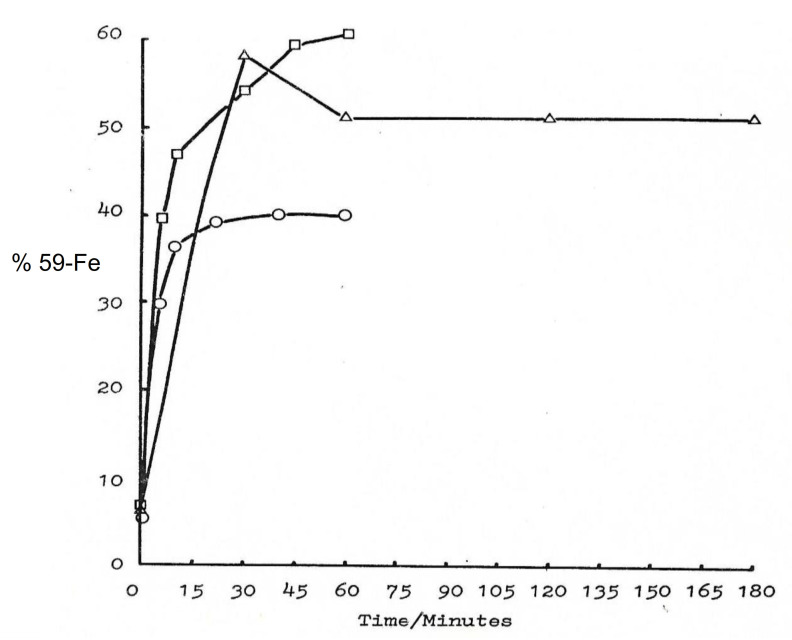
The transfer of iron by maltol in red blood cells. The rate of incorporation of the iron (59-Fe) in red blood cells was measured at different time intervals, following incubation with the iron (59-Fe) maltol complex at physiological pH. Three different experiments were carried out using different conditions including different buffers and variable maltol concentrations (0.5–4.0 mM) with molar excess ranging from 75 to 2800 times over iron [25].

The transfer of iron by maltol and other chelators in RBC was monitored using radioactively labelled iron (59-Fe). In a number of experiments under different conditions, the transfer of iron and incorporation in RBC by maltol proceeded rapidly and reached equilibrium within 30–40 min. About 40–60% of the iron (59-Fe) was incorporated in RBC and the remaining maltol iron (59-Fe) complex remained in the supernatant (Figure 6) [25].

By comparison and under the same conditions iron incorporation in RBC was less than 5% in the presence of the hydrophilic chelator iron complexes of deferiprone, deferoxamine, and 1,4-dihydroxypyridin-1-oxide (L3) (Kpar 0.02–0.05) (Table 4). In contrast, similar amounts of iron incorporation of about 50% in matured RBC to that of the maltol iron complex (Kpar 0.32) was observed with 2-hydroxy-4-methoxypyridine-1-oxide (L6) (Kpar 4.8) (Table 4) [25].

In much more extensive RBC studies of up to 3 h duration involving 19 chelators with a wide range of physicochemical properties, including Kpar of chelator iron complexes, several lipophilic iron complexes have been identified to be better transporters of iron into RBC than maltol [124]. The lipophilic iron complexes of tropolone, 8-hydroxyquinoline, and omadine with Kpar of 4.7, 10.0, and 2.7, respectively, caused an increased RBC uptake of iron (59-Fe) in the range of 80–90% by comparison to maltol iron (59-Fe) complex of about 50% (Table 4) [124]. Increased RBC uptake of iron (59-Fe) was also observed in similar experiments using lipophilic analogues of deferiprone [125].

It appears that, in general, lipophilic iron chelator complexes with neutral charge, including the maltol iron complex, readily diffuse across RBC membranes and incorporate iron in cellular compartments. In contrast, hydrophilic iron complexes, including those of deferiprone and deferoxamine, do not facilitate the transfer of iron across RBC membranes. Similar iron transfer properties using maltol and similar lipophilic chelator iron complexes are also expected in other cell types.

### 6.2. The Role of Chelators in the Uptake and Intracellular Distribution of Iron in Erythroid Cells

The transfer and distribution of iron in all the cells of the body under normal conditions is accomplished primarily by the plasma protein transferrin. The interactions of transferrin iron with chelators extracellularly and intracellularly appear to influence the intracellular distribution of iron. Furthermore, it is also expected that chelators could transfer iron in cells independently from transferrin and influence the intracellular distribution of iron.

Investigations regarding the iron metabolic pathways involved in the uptake and intracellular distribution of iron (59-Fe) from hydrophilic and lipophilic chelators of different physicochemical properties and transferrin iron have been carried out in erythroid cells in vitro. In studies using K562 cells or human bone marrow cells, iron (59-Fe) uptake into cells from lipophilic chelators, such as tropolone, 8-hydroxyquinoline, and maltol was many times higher than hydrophilic chelators, such as deferiprone, mimosine, and deferoxamine [126]. In particular, iron (59-Fe) uptake in bone marrow cells from these lipophilic iron complexes was about 40% times higher by comparison to transferrin iron (59-Fe) [126].

The uptake of iron by erythroid cells from lipophilic iron complexes was delivered in significant amounts in both haem and ferritin and none of the chelators has shown exclusive delivery to, or rejection of, a particular pathway, associated either with haem production or ferritin iron incorporation [126].

Similar results of iron uptake from lipophilic iron complexes were observed in a separate set of 24 h studies in K-562 and U-937 cells by 8 chelators, including maltol (0.6 mM) [127]. In addition to iron uptake in K-562 and U-937 cells, several other iron metabolic pathways were examined in the presence of chelators and their iron complexes, including transferrin membrane binding, DNA synthesis, and overall toxicity to cells, all of which appear to interfere with iron and other metabolic processes [127].

Transferrin iron uptake is usually regulated by the number of transferrin receptors present on the cell membrane of each cell [1,16,19,26]. Furthermore iron chelating drugs appear, in general, to cause reduced iron availability and increased transferrin receptor synthesis. In this context, 24 h incubation with most chelators, including maltol and deferiprone, but excluding the cytotoxic omadine and 8-hydroxyquinoline caused in both K-562 and U-937 cells lines, increased transferrin cell binding and, presumably, transferrin receptor synthesis. Similarly, under the same conditions, DNA synthesis was supported especially in the case of maltol [127]. The safety of maltol and the maltol iron complex was also confirmed in other cell viability and DNA synthesis studies using 14 chelators (0.02 mM) in the leukaemic cell lines K-562, U-937, HL60, and ML2. In contrast, under similar conditions, and even at lower concentrations, the lipophilic chelators and iron complexes of omadine, 8-hydroxyquinoline, and tropolone exhibited cytotoxic and DNA synthesis inhibitory effects [128].

It appears that chelators and chelator iron complexes cause variable effects on cellular iron metabolic pathways and, in some cases, selective cytotoxicity. In general, hydrophilic and moderate lipophilic chelator and chelator iron complexes, including maltol and the maltol iron complex, are less cytotoxic than lipophilic chelators and chelator complexes.

### 6.3. The Antimicrobial Effects of Maltol and Other Chelators

All living cells, including microbes, require iron for growth and development. Microbes synthesise specific chelators, termed siderophores, for the sequestration of iron from the surrounding medium [24,129,130,131,132,133]. The interaction of microbial cells with chelators and chelator iron complexes could, in principle, influence microbial growth. In this context, natural dietary chelators or drugs with chelating properties could act as siderophores by donating iron and promoting microbial growth [88,134,135]. In contrast, transferrin, lactoferrin, and other natural or synthetic chelators and chelator iron complexes could have the opposite effect and withdraw or prevent the uptake of iron from microbes, thus inhibit microbial growth [88,136,137,138].

In a number of experiments, the effect of maltol (0.35 mM) and the maltol iron complex on the growth of various types of bacteria in human serum was examined and compared to the chelating drugs deferoxamine (0.2 mM) and deferiprone, as well as four other KHP chelators (0.35 mM) [139]. Maltol was the only chelator that inhibited all four bacteria species, namely *Y. enterocolitica* (17%)*, S. epidermitis* (14%)*, E. coli* (39%), *and P. aeruginosa* (13%). In contrast, deferoxamine promoted the growth of three bacteria species (23–56%) and only inhibited *E. coli* (20%). By comparison, deferiprone inhibited the growth of three bacteria species (18–53%) and promoted the growth of *S. epidermitis* (10%) [139].

The effect of maltol was also investigated in relation to the growth of *P. falciparum* in a series of experiments for the identification of IND, to combat widespread multiple drug resistance in human malaria [140]. Maltol inhibited the growth of *P. falciparum* by about 15% at 0.01 mM and 50% at 0.10 mM by comparison to control. By comparison, deferiprone caused 52% and 87%, and deferoxamine 1% and 99% inhibition, using the same concentrations and conditions as maltol. Five other KHP chelators were also tested and showed to be more effective inhibitors (70–97%) than maltol at 0.10 mM [140].

Macrophages play a key role in iron metabolic processes, including the rapid turnover of iron due to the catabolism of effete RBC in the spleen and the liver, and in relation to antimicrobial activity. The effect of iron chelators on macrophage iron uptake and release could influence many processes, including antimicrobial activity. A study using mouse peritoneal macrophages and KHP chelators has shown that maltol enhanced iron mobilisation from macrophages pulsed with 59-Fe-transferrin-antitransferrin immune complexes [141]. Iron (59-Fe) release by maltol was higher than deferoxamine. Deferiprone and 1-ethyl-2-methyl-3-hydroxypyrid-4-one (L1NEt) were markedly more effective than deferoxamine, maltol, or mimosine. The release of iron (59-Fe) from macrophages increased with increasing chelator concentration above 0.008 mM and time of incubation from 2 to 14 h. None of the chelators appeared to donate significant amounts of iron to macrophages, and none showed any cytotoxic effects [141].

Overall it appears that maltol and its iron complex do not appear to promote microbial growth. Similarly, the macrophage studies suggest that the maltol iron complex, as well as other KHP complexes, do not appear to be recycled through the reticuloendothelial system [139,140,141].

### 6.4. Uptake of Iron Maltol by the Enterocyte

Maltol and other lipophilic chelators were first identified as potential IND for increasing iron absorption in the original RBC and rat jejunum permeation screening studies of phytochelators and synthetic chelators, with potential for clinical use in IDA. The investigations were carried out at the same time as the screening of KHP and other chelators, which were intended for the treatment of iron overload and other diseases of iron metabolism. The latter investigations led to the identification of the iron chelating drug deferiprone, which has been used in iron loaded patients since 1987 [25,142,143].

In the in vitro rat jejunum permeation screening studies, the lipophilic KHP chelators selected for investigation were used at a six molar ratio excess over iron (0.01 mM). In these studies maltol and L6 were identified to be 2.5 and 5.0 times more effective than the phytochelators catechol and citrate, respectively, in the transfer of iron (59-Fe) across the inverted jejunum sac. Furthermore, in the same studies, the L6 iron (59-Fe) complex was shown to be partitioning in the intestinal tissue to a greater extent than the maltol and also the other iron complexes [25].

Increased iron uptake across the small intestine of the rat from the maltol iron complex was observed in confirmatory subsequent studies [143]. Overall, it appears from the original rat jejunum permeation studies in vitro that maltol and other lipophilic chelator iron complexes can be taken by the intestinal tissue and transferred across the intestinal wall, thus facilitating iron absorption [25,143].

## 7. In Vivo Studies of the Effect of Maltol and Other Chelators on Iron Absorption

Under normal conditions, the rate of iron absorption is controlled in the enterocyte by a regulatory pathway involving ferroportin and hepcidin (Figure 1) [1,2,3,4,43,44,45,46,47,48]. However, under certain conditions, e.g., in African siderosis and accidental iron poisoning, the normal regulatory pathway appears to be overwhelmed and cannot control or influence the increased rate of gastrointestinal iron absorption [62,63,64]. Similarly, the rate of iron absorption appears to be influenced by the presence of iron chelators and other factors, which are separate from the regulatory pathway involving ferroportin and hepcidin [23,43,44,45,46,47,48].

### 7.1. Increase in Iron Absorption in Animals Using Maltol and Other Lipophilic Chelators

Natural and synthetic iron chelating compounds are known to influence the absorption of iron and some of them may have a use in the treatment of diseases associated with gastrointestinal iron absorption imbalance, which may lead to IDA or iron overload, e.g., idiopathic haemochromatosis and thalassaemia intermedia [23,28,42,54,55,56,57,58].

Following the original investigations and identification of chelators for potential use in the treatment of IDA, an in vivo screening system was developed using chelator iron (59-Fe) complexes uptake and body distribution in mice [25,144]. In this context, the effect of 17 natural and synthetic chelators on iron (59-Fe) absorption in mice has been investigated in three different experiments using single and repeated intragastric administrations of chelator iron (59-Fe) complexes [144].

The amount of iron (59-Fe) in whole animals, their excretions, and body distribution of iron (59-Fe) in blood, liver, spleen, and heart was measured at one, three, and eight weeks following the iron (59-Fe) chelator mixture intragastric administrations and compared to controls, which received the same amount of iron (59-Fe), but no chelator. The mixture contained several times excess chelator over iron. The amount of iron (59-Fe) absorbed was less than 3.5% of the total iron (59-Fe) administered. The 2-hydroxy-4-methoxypyridine-1-oxide (L6) and maltol, which form neutral, lipophilic iron complexes, were found to cause a significant increase in 59-Fe absorption, while hydrophilic chelators including deferoxamine, deferiprone, mimosine, and EDTA caused a significant decrease in iron (59-Fe) absorption by comparison to controls. It was also observed that under the same conditions, 2-hydroxypyridine-1-oxide (L4), 8-hydroxyquinoline, and omadine caused iron precipitation, which was not absorbable. In general, several factors, including chelator concentration, water solubility of the chelator and its iron complex, and food composition, appear to affect iron absorption. The overall in vivo results in mice were in broad agreement with the in vitro results of the RBC and rat inverted intestine experiments, where lipophilic chelators have been shown to increase the transfer of iron across cell membranes and across the gastrointestinal tract, thus increasing iron absorption [25,143,144].

Monitoring of the iron (59-Fe) body distribution in mice following the oral administration of chelator iron mixtures has also shown that most of the iron absorbed from the chelator iron mixtures was detected in RBC and less in the organs, where it was mainly distributed in the liver (50–60%), compared to the spleen (30–40%) and heart (10%>). Overall, the findings suggested that the iron (59-Fe) absorbed following the oral administration of chelator iron complexes is diverted primarily to the bone marrow and utilised for the production of haemoglobin [144].

Similar results of increased iron (59-Fe) uptake were observed following the intraduodenal administration of the (59-Fe) iron maltol in rats, by comparison to other hydrophilic iron (59-Fe) mixtures with EDTA, gluconate, and fumarate [145]. Dissociation of iron from maltol was suggested following entry in the intestinal wall with each molecule taking a different metabolic pathway [146]. The intravenous administration of an (59-Fe) iron maltol mixture in rats also resulted in the dissociation of iron and maltol, presumably through donation of iron to transferrin and release of maltol [146]. In all rat study cases, iron (59-Fe) uptake was mostly associated with haemoglobin production and less with liver deposition and storage. The increased uptake of iron (59-Fe) in the presence of maltol and its utilisation by the haematopoietic tissues has been observed in both mice and rats, despite the suggested dissociation of iron and maltol, and the kinetic variations in iron uptake following the use of different iron concentrations in the mixture [146].

Prolonged intragastric administration of lipophilic chelators, such as 8-hydroxyquinoline, have been previously shown to cause iron overload in animals by mobilising and transferring iron present in food across the gastrointestinal tract [147,148]. For example, the 18-month administration of 8-hydroxyquinoline in animals caused haemosiderosis initially in the liver and spleen, and eventually in the heart, kidneys, adrenal glands, pancreas, testes, and thyroid [147]. It is envisaged that lipophilic natural dietary chelators, such as maltol or synthetic drugs (such as deferasirox) could also increase dietary iron absorption [23,36,42]. In this context, these and other lipophilic chelators could be used for long term administration, promoted as food additives or supplements, and in food fortification for facilitating iron absorption and the prevention of IDA [23].

### 7.2. The Effect of Maltol and Maltol Iron Complex on Iron Excretion

The pharmacological and toxicological properties of the chelators involved in the iron complex formulations are critical parameters for the safety and efficacy used in the selection process for identifying IND for the treatment of IDA [28]. For example, a serious toxic side effect by the chelator, its metabolites, or the iron complex could minimise the prospects of the regulatory approval of the iron complex formulation for clinical use. Similarly, if the iron of the iron complex formulation is absorbed, but rapidly excreted unchanged, or is not utilised by the haematopoietic tissues, then it cannot be considered as a potentially effective IND treatment for IDA [28].

In the case of the maltol iron complex formulation and most of the other lipophilic chelator iron complexes, iron has been shown to be absorbed and utilised by the haematopoietic tissues and also for storage in ferritin [144]. Free unbound maltol dissociated of iron after iron uptake enters the blood stream, metabolised and excreted. In this context, the effect of the dissociated maltol on iron excretion is essential information for determining the iron balance following the administration of the trimaltol iron complex formulations. In the original studies of discovery of the maltol iron complex for potential use in IDA, maltol has also been investigated for possible toxicity and also efficacy in iron removal in mice [25].

**Figure 7:**
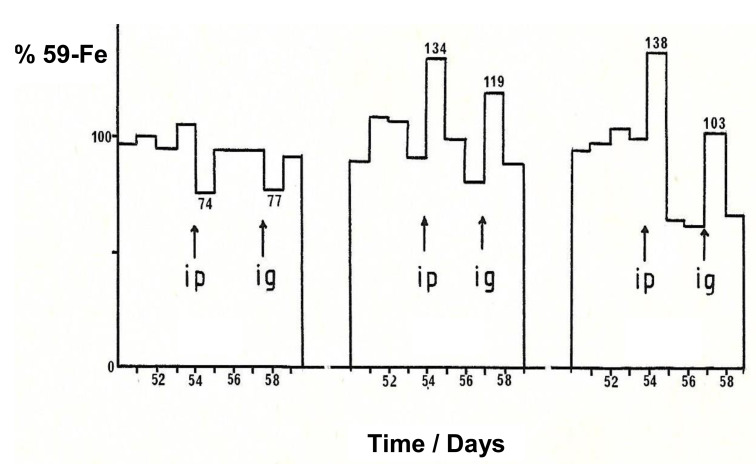
The effect of maltol on daily iron excretion in mice. The profile of iron (59-Fe) excretion in iron loaded, 59-Fe labelled mice following treatment with maltol (250–300 mg/kg). Maltol was administered in three mice initially intraperitoneally (ip) and after 3 days intragastrically (ig), as indicated by arrows. In each mouse, the mean iron (59-Fe) excretion 3–4 days prior to the recorded values was considered as the background (100%) excretion of (59-Fe) [25].

No toxicity was observed using maltol at intraperitoneal doses of 500 mg/kg in mice. In the iron (59-Fe) metabolic studies in mice using intraperitoneal (7 mice) and intragastric (3 mice) administrations of a dose of 250–300 mg/kg, no significant increase in iron excretion was observed with maltol by either route (Figure 7). Under the same conditions using deferiprone intraperitoneally and intragastrically, the increase in iron (59-Fe) excretion was about 270–290% higher than controls and in the case of deferoxamine about 340% higher when administered intraperitoneally, but not intragastrically [25].

In contrast to maltol, the lipophilic oral iron chelating drug deferasirox causes both an increase in dietary iron absorption and an increase in iron excretion in iron loaded animals and patients [42,58,79,149,150,151]. However, at the high doses (20–40 mg/kg) used in iron loaded patients, the level of iron excretion is much higher than the limited amounts of available dietary iron absorption caused by the oral administration of deferasirox [58,79,149,150,151].

In general, it is anticipated that maltol dissociated from iron following the oral maltol iron mixture formulation administration is not likely to cause increases in iron excretion. Furthermore, the concentration of maltol in the maltol iron formulation dose is very low for dissociated maltol to cause negative iron balance. Similarly, the prospects of toxicity are also very low because of the low dose of maltol by comparison to the dose of the other chelating drugs used in the treatment of iron loaded patients and in other conditions with normal iron stores [152,153,154,155,156,157,158,159].

## 8. Factors Affecting the Absorption of Maltol and the Maltol Iron Complex

Many factors can affect the rate of maltol iron complex absorption and body iron distribution in vivo and in therapeutic responses for different categories of patients. Some of these factors include variations based on individual absorption, distribution, metabolism, excretion, and toxicity (ADMET) response profiles, and pharmacogenomic, proteogenomic, metabolomic, redoxomic, and metallomic factors [28,42,79]. Additional factors related to chelator or chelator metal complex absorption include competition from other metal ions, dietary molecules and drugs, reducing agents, pH in the gastrointestinal tract, etc. (Table 1) [28,42,160].

In considering the various factors affecting the level of iron absorption following the oral administration of the maltol iron complex mixture, of major importance is the quantity of iron present in the formulation mixture. In addition, dissociated maltol of the iron formulation mixture could also facilitate the absorption of non-haem dietary iron present in the gastrointestinal tract.

Many dietary molecules and medicinal drugs could also interact with maltol and the maltol iron complex and influence the absorption of iron. The presence of dietary molecules with chelating properties such as tannins and phosphates in the gastrointestinal tract will compete with maltol for iron and inhibit its absorption [160,161,162,163,164]. In contrast, lipophilic phytochelators, such as 8-hydroxyquinoline and omadine, could act synergistically and enhance the absorption of iron from the maltol iron complex mixture [147,148]. Similar interactions and influence in the absorption of iron is also anticipated in the presence of drugs with chelating properties. For example deferoxamine, deferiprone, hydroxyurea, penicillamine, bisphosphonates, levodopa, mycophenolate, and tetracycline could interact with the maltol iron complex and inhibit its absorption, whereas deferasirox is anticipated to promote its absorption [54,55,56,59,60].

Synergistic effects for increasing iron absorption by maltol and the maltol iron complex is anticipated in the presence of reducing agents, such as the chelator antioxidant vitamin ascorbic acid [52]. In contrast, metal ion competitors for binding maltol, such as zinc and copper, could displace iron from the maltol iron complex and decrease gastrointestinal iron absorption [165]. Under normal conditions the inhibition of iron absorption is proportional to the concentration of the competing metal ion [53]. Similar inhibitory effects are expected in the presence of aluminium and other metal ions, which also have high affinity for maltol, other similar chelators, and transferrin [166,167,168]. In this context, the co-administration of aluminium based antacids or other metal ion mixtures are not recommended for the treatment of IDA with maltol iron complex formulations [53].

All the above anticipated molecular interactions are subject to thermodynamic and kinetic parameters and require further investigations. The formation of ternary complexes of maltol and the maltol iron complex with other chelating molecules, similar to the deferiprone and ascorbate ternary iron complex, is particularly important for assessing the promotion or inhibition of iron absorption [61].

Many other factors and conditions from physicochemical molecular changes to clinical variations, due to the underlying conditions in each disease and patient, could also influence the absorption of maltol and the maltol iron complex. For example, the quantity of water, alcohol, and other fluid intake, as well as dietary factors affecting the solubilization of maltol and the maltol iron complex in the formulation could affect iron absorption. Similarly, the pH of the stomach and intestine, as well as the use of antacids and other drugs and dietary molecules affecting iron transport across the enterocyte, e.g., Nifedipine, which is an L-type calcium channel blocker, could also affect the absorption of iron (Table 1) [23,36,42,160].

Changes in the normal function of the gastrointestinal tract and the haematopoietic tissues is observed in many diseases, including iron metabolic diseases, anaemia of chronic disease in cancer and inflammation, haemoglobinopathies, nephropathy, etc., all of which can affect iron absorption and distribution [23,57,58,169,170]. Similar effects are observed in diseases with abnormal organ function, gastrectomy, and other interventions where gastrointestinal tract function is affected, and also in ageing [23,36,42]. In all these cases, the rate of absorption and utilisation of iron from different iron formulations, including the maltol iron complex, is expected to be affected.

## 9. Clinical Studies Related to the Efficacy of Iron Maltol in Iron Deficiency Anaemia

The preclinical and clinical development of the maltol iron complex in animal and clinical studies continued intermittently for many years and by different groups of investigators following its discovery [25]. Many studies are still ongoing for different categories of patients with IDA conditions. In almost all the clinical studies and trials, oral trimaltol iron has been shown to be tolerable and effective, with significant improvements in reversing anaemia and normalising serum iron parameters in different categories of patients with IDA. The most common adverse effect during the clinical studies was mild gastrointestinal intolerance [171,172].

In pharmacokinetic and metabolic randomised phase I study, ferric maltol, and its effect on iron indices has been evaluated in 24 iron deficient patients with inflammatory bowel disease (13 with Crohn’s disease and 11 with ulcerative colitis) [173]. The patients received oral ferric maltol 30, 60, or 90 mg twice daily during an 8 day period. Rapid absorption was observed following administration, with plasma maltol and maltol glucuronide increasing rapidly at all doses in a dose proportional pattern and reaching maximum plasma concentration 1.0–1.5 h post-dose and declining to baseline after 3–6 h. No maltol accumulation was observed after 7 day dosing. Serum iron and transferrin saturation increased with all doses reaching maximum values at 1.5–3.0 h post-dose. Similarly, serum ferritin and reticulocyte haemoglobin content increased by the eighth day, with greater improvements at the higher doses [173]. The ferrokinetic, pharmacokinetic and metabolic properties of maltol appear to be very similar to that previously observed with the oral chelating drug deferiprone [91,92].

The efficacy of maltol iron for the treatment of IDA was demonstrated in many other clinical studies and trials. In one study involving 23 patients non-tolerant to ferrous sulphate, maltol iron containing 30 mg iron was taken for 3 months twice daily before breakfast and the evening meal. Nineteen of the 23 patients completed the study and anaemia was fully corrected in 14 of these with mean haemoglobin increasing from 106 to 126 g/L and also significant increase in serum ferritin from 8.1 to 17.4 μg/L [174].

In another clinical comparative study, maltol iron administration has been shown in 20 IDA individuals to be both more rapid and total absorption greater than that seen with ferrous sulphate. These results were observed at two different doses of 10 and 60 mg elemental iron tablet formulations [175]. In general, iron from the maltol iron formulation treatment appears to be at least as well absorbed as from the ferrous sulphate formulation, suggesting that ferric maltol was the first ferric iron formulation to be absorbed to a degree equivalent to that of ferrous iron salts [175].

In a phase III clinical trial, randomised, double-blind, placebo-controlled study design, which included patients with inflammatory bowel disease, maltol iron administration (30 mg, twice daily) for up to 12 weeks increased haemoglobin levels of the treated patients in comparison to placebo treated patients. Significant increases of haemoglobin were observed at 4, 6, and 12 weeks after treatment and in two-thirds of the patients’ haemoglobin levels normalised at the end of the 12 week period. No toxic side effects were reported and the safety profile was comparable to placebo. Furthermore, the 12-week treatment had no impact on inflammatory bowel disease severity [176]. This study suggested that maltol iron can be used as an alternative to intravenous iron and other iron formulations for the treatment of IDA patients with inflammatory bowel disease [176]. Similar results of the efficacy and safety of maltol iron were obtained in several other studies involving IDA patients with inflammatory bowel disease [170,171,172,174,176,177,178].

Several clinical trials in other conditions with IDA, including chronic kidney disease and pulmonary hypertension, have confirmed the high efficacy and low toxicity of maltol iron [169,171,172,179,180]. For example, the use of the approved dose of 30 mg twice daily of maltol iron for 12 weeks in 20 pulmonary hypertension patients with IDA caused an increased in the haemoglobin levels from 10.7 to 13.6 g/dL, in transferrin saturation from 7.5% to 31.5%, and serum ferritin from 13.1 to 33.6 μg/L [179]. The treatment with maltol iron was well-tolerated over the whole 12-week period.

There are several advantages in selecting trimaltol iron formulation over other iron formulations for the treatment of IDA. It is generally effective, safe and more selective in iron delivery than other formulations because of its lipophilicity, physiochemical, pharmacological, and other properties [25,78,79,171,172,174]. The selectivity for iron delivery by maltol formulations decreases the toxicity associated with the excess, non-absorbable iron observed with other iron formulations. Furthermore, the high efficacy and low toxicity of maltol iron formulations could benefit many patients experiencing complications and toxicity with intravenous iron, ferrous sulphate and other iron formulations [181,182,183,184,185,186].

In considering individual patient variations on responses, such as ease of administration, low toxicity and timing of the normalisation of haemoglobin, maltol iron formulations may also appear to have further advantages over the other iron formulations. The cost of maltol iron and worldwide accessibility to patients is another major issue affecting global health strategies, especially since the vast majority of IDA patients live in developing countries. However, the cost of maltol and iron are negligible and many other maltol iron formulations can be prepared and considered as off-patent or generic pharmaceuticals [69,79]. Overall, maltol iron has the prospects of becoming the first choice for worldwide treatment of patients with IDA.

## 10. Potential Applications of Maltol and Maltol Metal Complexes in Medicine

Despite that the discovery and characterisation of maltol and other novel KHP chelators and their metal complexes, as well as their potential for clinical uses was identified about 40 years ago, the progress regarding their pharmaceutical development was very slow [25,69]. In the case of maltol, the pharmaceutical development process could have been rapid and inexpensive, considering that it was a widely available natural product and food component, which has been used for more than 50 years.

There are many prospects for the use of iron and other metal complexes of maltol. One new therapeutic approach is the potential use of maltol in food and drink fortification, which may be considered as an additional method for treating IDA, considering that maltol can facilitate the absorption of non-haem dietary iron present in the gastrointestinal tract. The slow increase of dietary iron uptake over long-term periods using food products fortified with maltol may benefit millions of IDA patients worldwide and especially in developing countries where IDA is a major health problem. This approach is anticipated to be less toxic than the administration of established iron formulations where most of the iron is not absorbed causing toxicity in the gastrointestinal tract (Table 3) [12,13,14,184,185,186].

Maltol and other lipophilic chelators, as well as their metal complexes, could be used for many other applications and areas of medicine [28,53,187,188,189,190,191,192,193,194,195,196]. Some of these applications include the treatment of other essential metal deficiency or related diseases, such as diabetes, delivery of redox active metal complexes against cancer cells and delivery of xenobiotic metals to disrupt essential metal, e.g., iron pathways in cancer, diagnostic metal delivery in diseases, including inflammation and cancer, radiolabelling of cells using metal radiotracers, and finally, theranostic metal delivery in different diseases [28,53,187,188,189,190,191,192,193,194,195,196].

The ability of maltol to bind different metal ions and to form lipophilic metal complexes, its interaction with transferrin and metal transfer ability intracellularly, as well as its low toxicity make it an ideal candidate for many clinical applications in addition to its iron complex role for the treatment of IDA [11,28,88]. The affinity of maltol for many other metal ions in addition to iron such as copper, zinc, aluminium, gallium, indium, gadolinium, ruthenium, chromium, and vanadium is similar to (but lower than) that observed with transferrin and deferiprone [88,187,188,189,190,191,192,193,194,195,196,197]. Within this context, the theranostic and diagnostic fields associated with the xenobiotic metal complexes of maltol involve many diseases and applications which are continuously expanding and evaluated [192,193,194,195,196,197].

One major medical application of maltol is related to the trimaltol gallium complex (gallium maltolate), which is one of the most developed maltol metal complexes that have reached the stage of clinical trials and veterinarian use mainly for anticancer and antimicrobial activity [197,198,199,200,201,202,203,204,205,206,207,208,209,210]. The maltol gallium complex of maltol 3:1 gallium stoichiometry is neutral, similar to the trimaltol iron complex but is slightly more lipophilic, with the n-octanol/water partition coefficient of 0.40 versus 0.32 of the trimaltol iron complex.

The basic anticancer and antimicrobial targeting of maltol gallium is the delivery of gallium to cells which mimics iron and affects iron-related metabolic pathways. In this context, the antimicrobial activity of trimaltol gallium is thought to be based on the substitution of iron by gallium causing the deprivation of iron, which is essential for the rapid growth of pathogenic microbes [130,131,132,133,203,204,205,206,207,208,209,210]. The anticancer activity of trimaltol gallium is thought to be based on the delivery of gallium to transferrin causing reduction of cancer cell acquisition of iron and growth reduction through the slow turnover of the iron dependent enzyme ribonucleotide reductase leading to the inhibition of DNA synthesis [28,88,188,196,197,198,199,200,201,202]. In particular, cancer types, such as breast, prostate, bladder, and leukaemia with abundance of transferrin receptors and upregulated production of ribonucleotide reductase render such tumour cells susceptible to the cytotoxicity of gallium [28,88,188,196,197,198,199,200,201,202,211].

Another major, rapidly expanding area of the application of maltol and other chelators in medicine is in the diagnostic and theranostic fields [28]. The design and targeted use of different chelating agents, which form metal complexes of variable physicochemical properties, appear to affect the bodily distribution of specific metal radiotracers and are increasingly applied for the diagnosis, as well as progress tracking of different diseases [28,212,213]. Similar targeted chelator metal complexes have been identified for the theranostic application of radiotracer metals in inflammation, cancer, and other diseases [28,212,213]. For example, it has been shown that, in the case of trimaltol gallium (67-Ga) complex in hepatocellular carcinoma patients, the cancer was highly gallium-avid in a (67-Ga) diagnostic scan and was treated with oral trimaltol gallium complex at 4 doses of 1.5 g/day for four weeks. Significant improvements of cancer indices were observed and necrosis of the tumour has been shown by CT scans at eight weeks [202].

There are many other areas of potential investigations and applications in medicine involving maltol and maltol metal complexes. In each case, specific conditions are applied and appropriate protocols need to be designed. For example, intravenous trimaltol iron complex formulations may have to be designed to benefit IDA cancer and other categories of patients [28,214,215,216]. Mixed chelator iron complexes of maltol and ascorbate may also be designed for increasing the efficacy of gastrointestinal iron uptake for the treatment of IDA. Similarly, the use of other metal complexes may also result in the improvement of diagnostic and theranostic applications, as well as in synergistic effects with drugs or proteins in the treatment of different diseases [28,52,61,217,218,219,220].

## 11. Conclusions

Iron deficiency anaemia affects billions of patients worldwide, including children, pregnant women, vegetarians, malnourished individuals, as well as patients with cancer, inflammatory bowel disease, chronic kidney disease, and many other diseases. There are many ferric and ferrous iron formulations available for the treatment of IDA, but most of these are not sufficiently effective and cause toxic side effects, mainly because of the excess iron administration.

The identification and characterisation of the in vitro and in vivo properties of maltol and the trimaltol iron complex has instigated the clinical investigations and development of new pharmaceuticals for use in iron deficiency and several other areas of medicine. In particular, as a result of the clinical approval of the trimaltol iron complex significant therapeutic improvements have been observed in different categories of patients with IDA, increasing the prospects of introducing more effective and less toxic treatments for millions of patients worldwide.

The specificity of the alpha-keto hydroxy binding site of the KHP chelators for iron, the neutral charge, and the lipophilicity of maltol and its iron complex are some of the molecular characteristics that contributed to the development of this new class of pharmaceuticals. Similar molecular characteristics have been observed in 2-hydroxy-4-methoxypyridine-1-oxide (L6), tropolone, and 8-hydroxyquinoline, which have also been shown to increase iron absorption in animals, and the same—or their analogues—may also be developed for the treatment of IDA.

The development of maltol and other naturally occurring lipophilic iron chelators as nutraceuticals, and in food fortification, may increase the prospects of facilitating the treatment of IDA in many categories of patients, including malnourished and poor patients in developing countries.

Several other possible clinical applications of metal maltol or other lipophilic metal complexes are currently developed, such as metal radiotracers in diagnostic medicine, theranostic formulations, and anticancer formulations. In particular, the clinical evaluation of trimaltol gallium in cancer patients increases the prospects of development of a new class of therapeutics in cancer, infectious diseases, and other diseases.

Further studies are required for identifying the mode of action and effects of maltol iron and other lipophilic iron complexes on iron metabolism at the molecular and subcellular levels, and other levels. In particular, the interactions of maltol and maltol iron complex with proteins of iron metabolism are very important in the turnover and inhibition/activation of enzymes, such as ferroportin and hepcidin involved in metabolic pathways associated with iron absorption, and other normal bodily functions. Similarly, further studies are required for the evaluation of the interactions among maltol xenobiotic metal complexes with iron metabolic pathways, and other pathways affecting their metabolism, toxicity, and therapeutic actions.

## Figures and Tables

**Table 1:** Non-Regulatory Factors Affecting Iron Absorption.

**Quantitative aspects** The quantity of iron present in the diet, e.g., excess iron intake in African siderosis or insufficient iron intake in vegetarian populations
**Qualitative aspects** The form of dietary iron present in food, e.g., haem, ferrous, ferric, ferritin, hemosiderin
**Role of reducing agents** The presence of reducing agents, e.g., ascorbic acid converts Fe (III) to Fe (II) and increases iron absorption
**Effect of dietary molecules** The presence of dietary molecules with chelating properties, e.g., phytates and tannins decrease iron absorption
**Effect of drugs with chelating properties** The presence of drugs with chelating properties, e.g., deferiprone, deferoxamine, tetracycline, hydroxyurea inhibit iron absorption
**Effect of fluids** The quantity of water, alcohol, and other fluid intake can influence the dissolution of iron supplements or other components in the gastrointestinal tract
**Effect of molecules affecting cellular iron transport** Dietary molecules and drugs affecting iron transport across the enterocyte (e.g., Nifedipine, which is an L-type calcium channel blocker)
**Dietary factors affecting iron solubilisation** Dietary factors affecting the solubilization or precipitation of iron. Insoluble iron is not readily absorbed
**Effect of pH on iron solubilisation** pH of the stomach and intestine, e.g., the higher the pH the lower the solubility of iron; Antacids decrease iron absorption
**Anatomical changes and iron absorption** Gastrectomy and other surgical interventions, which can affect gastrointestinal iron absorption. Body weight, e.g., obese people absorb less iron than normal body weight individuals
**Effect of diseases on iron absorption** Malignancy, infectious and other diseases, haemoglobinopathies
**Changes in iron absorption levels** Malnutrition, food poisoning, ageing

**Table 2:** Examples of anaemias treated with iron supplements.

Iron deficiency anaemia due to increased iron requirements (e.g., pregnant and menstruating women, young children)
Iron deficiency anaemia due to insufficient dietary iron (e.g., vegetarian populations, malnutrition)
Anaemia of chronic disease or anaemia of inflammation, in neoplastic, infectious and inflammatory diseases, mainly in cases with concurrent iron deficiency or in cases of combination with erythropoietin treatment (e.g., Inflammatory bowel disease, cancer, rheumatoid arthritis)
Chronic kidney disease including haemodialysis patients
Chronic cardiac failure

**Table 3:** Examples of iron complexes used for the treatment of iron deficiency anaemia.

**Ferrous iron formulations** (Ferrous sulphate, ferrous ascorbate, ferrous fumarate, ferrous gluconate, ferroglycine sulphate
**Ferric iron formulations** (Ferric fumarate, ferric polymaltose, iron dextran, ferric gluconate, ferric iron sucrose, ferric saccharate, iron bis-glycinate chelate
**Ferric intravenous iron formulations** (Iron sucrose, ferric carboxymaltose, ferric gluconate, ferumoxytol, iron isomaltoside-1000, iron dextran (low-molecular-weight forms)
**Lipophilic (hetero)aromatic iron complex formulations** (Ferric maltol, ferric 8-hydroxyquinoline, ferric tropolone, ferric 2-hydroxy-4-methoxypyridine-1-oxide (L6)

**Table 4:** Physicochemical properties of chelators and their iron complexes.

Chelator	Log β	MWt	Kpar	Charge	Kpar Iron	Charge Complex
Maltol	30	126	1.23	neutral	0.32	neutral
Tropolone	32	122	3.04	neutral	4.50	neutral
8-Hydroxyquinoline	37	145	28.30	neutral	10.00	neutral
L3	30	127	0.09	zwitterionic	0.04	neutral
L4	NA	111	0.09	zwitterionic	0.95	neutral
L6	29	155	0.37	zwitterionic	4.85	neutral
Omadine	NA	127	0.04	zwitterionic	2.67	neutral
Mimosine	36	198	0.01	zwitterionic	0.01	zwitterionic
Deferoxamine	31	561	0.02	positive	0.02	positive
Deferiprone	35	139	0.18	neutral	0.05	neutral
Deferasirox	27	373	6.30	negative	NA	negative

Iron complex stability constants (log β); molecular weight (MWt); n-octanol/water partition coefficients (Kpar); charge of chelator and chelator iron complex at physiological pH (charge); 2,4-dihydroxypyridine-1-oxide (L3); 2-hydroxypyridine-1-oxide (L4); 2-hydroxy-4-methoxypyridine-1-oxide (L6). Not available (NA).

## Data Availability

Not applicable.

## Abbreviations

2,4-Dihydroxypyridine-1-oxide	(L3)
2-Hydroxypyridine-1-oxide	(L4)
2-Hydroxy-4- methoxypyridine-1-oxide	(L6)
Diferric transferrin	(Fe-T-Fe)
Divalent metal transported protein	(DMT1)
Ethylenediaminetetraacetic acid	(EDTA)
Optical density	(OD)
Hydroxyeicosatetraenoic acid	(HETE)
Hydroperoxyeicosatetraenoic acid	(HPETE)
Investigational new drugs	(IND)
Iron deficiency anaemia	(IDA)
Red blood cells	(RBC)
